# Self-Reported Hindering Health Complaints of Community-Dwelling Older Persons: A Cross-Sectional Study

**DOI:** 10.1371/journal.pone.0142416

**Published:** 2015-11-16

**Authors:** Sophie C. E. van Blijswijk, On Ying A. Chan, Anne H. van Houwelingen, Jacobijn Gussekloo, Wendy P. J. den Elzen, Jeanet W. Blom

**Affiliations:** Department of Public Health and Primary Care, Leiden University Medical Center, Leiden, The Netherlands; Medical University Vienna, AUSTRIA

## Abstract

**Purpose:**

Proactive care for community-dwelling older persons targeting self-reported hindering health complaints might prevent a decline in function. We investigated the spectrum of self-reported hindering complaints of community-dwelling older persons, the association with functional outcomes, and help-seeking behavior for these complaints.

**Methods:**

Within the ISCOPE trial, participants (aged ≥75 years) received the ISCOPE screening questionnaire, including the open-ended question “*At the moment, which health complaints limit you the most in your day-to-day life?*”. After coding the answers with the ICPC-1-NL, we examined the prevalence and the association between the number and type of complaints and functional outcomes (Groningen Activities Restriction Scale, quality of life measured on Cantril’s Ladder, Mini-Mental State Examination, Geriatric Depression Scale-15, and De Jong Gierveld Loneliness Scale). Electronic patient registers were searched for the most reported complaints.

**Results:**

7285 participants (median age: 81.0 years [IQR 77.8–85.3], 38.6% males) reported 13,524 hindering complaints (median 1, range 0–18); 32.7% reported no complaints. Participants mostly reported problems with walking/standing (22.1%), pain (20.8%) or weakness/tiredness (8.5%). These complaints were mentioned in the electronic patient registers in 28.3%, 91.3% and 55.5%, respectively. Higher numbers of hindering complaints were related to poorer scores on the number of domains with problems, Cantril’s Ladder for quality of life, Groningen Activities Restriction Scale, Geriatric Depression Scale, and De Jong Gierveld Loneliness Scale. Self-reported weakness, problems with walking/standing, visual limitations, cognitive problems, dyspnea and back complaints were associated with poorer scores on the number of domains with problems, Groningen Activities Restriction Scale, MMSE or Geriatric Depression Scale.

**Conclusion:**

One third of the participants reported no hindering complaints. Problems with walking/standing, pain, and weakness/tiredness were most reported, but not always found in electronic patient registers. A higher number of, and specific self-reported hindering complaints, were associated with poorer scores on functional outcomes. It may be helpful for general practitioners to ask about these complaints and their influence on daily life.

## Introduction

Healthcare professionals face the challenge of continuously providing adequate healthcare to an ever-growing older population [1, 2] with, generally, a higher level of disability and dependency. Prevention of the start and progress of disability, thereby preventing dependency on caregivers, is an important part of this challenge [3–7].

The presence of health complaints, such as impaired mobility, urinary incontinence, and depressive symptoms, is negatively associated with the ability to function independently and quality of life (QoL) [8–14]. This effect is enhanced when several problems are present simultaneously, which is not uncommon in older persons [15]. Unfortunately, the presence of these health complaints often remains unknown to the general practitioner (GP) until these complaints have led to a decline in functional status and an increase of dependency [16]. As previously shown, the agenda of the GP in asking older persons for unmet needs is often different from the agenda of the older person himself/herself [17]. To effectively shape proactive care, healthcare providers need to know which health complaints most hinder older persons from day-to-day.

Therefore, we investigated the spectrum of self-reported hindering health complaints of community-dwelling older persons. In this cross-sectional study, we examined health complaints that older persons reported as being the most limiting in their daily life. Their help-seeking behavior related to these complaints was evaluated by searching the electronic patient records for these complaints. We evaluated the association between the number of self-reported hindering complaints and the presence of the ten most reported hindering complaints with several functional outcomes.

## Methods

### Study design and population

This cross-sectional study is embedded in a cluster-randomized trial: the ISCOPE (Integrated Systematic Care for Older Persons) study (Netherlands trial register, NTR1946). In short, the aim of the ISCOPE study was to assess the cost-effectiveness of the identification of older persons with complex problems and to organize healthcare in a care plan. The ISCOPE study included 59 general practices. Patients with a life expectancy of less than three months were excluded from the study, as were nursing home residents, those considered too ill by the GP, non-Dutch speaking patients, and other patients based on additional reasons provided by the GP. All other persons aged ≥75 years (n = 11,476) were invited to participate.

**Fig 1:**
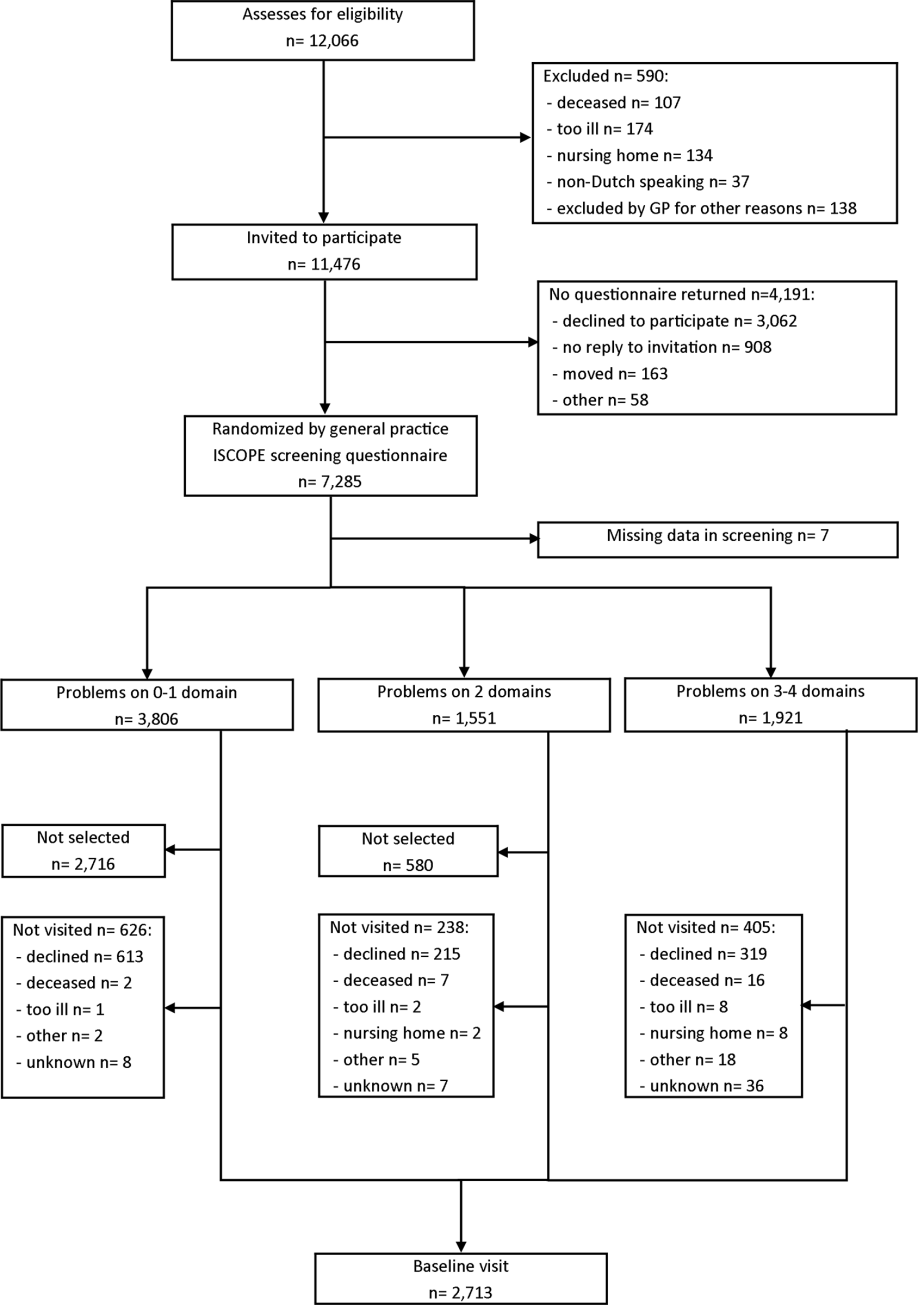
Flowchart ISCOPE.

Together with the invitation, they received the ISCOPE screening questionnaire by mail (S1 Appendix). Participants were asked to complete the questionnaire and return this by mail. After three weeks, participants who did not return the questionnaire were contacted by telephone by a research nurse and were offered help (by telephone, or face-to-face) to complete the screening questionnaire. The ISCOPE screening questionnaire covers four health domains: functional, somatic, psychological, and social. All participants with problems on three or four domains were visited by research nurses to obtain information on socio-demographic characteristics and on functional outcomes with validated questionnaires. For practical reasons, a random selection of 15% of participants with problems on zero or one domain, and 60% of participants with problems on two domains were visited (Fig 1). The inclusion period was from September 2009 until September 2010. The Medical Ethical Committee of the Leiden University Medical Center approved this study. All participants gave informed consent [18].

### ISCOPE screening questionnaire

The ISCOPE screening questionnaire includes 21 closed-ended questions on problems of the functional, somatic, psychological, and social domain (S1 Appendix). Examples of the questions are “*How well would you say you cope with your general day-to-day life*?” and “*Are you using more than four different kinds of medicine at the moment*?”.

The ISCOPE screening questionnaire concluded with the open-ended question “*At the moment*, *which health complaints limit you the most in your day-to-day life*?” to reveal which hindering complaints older persons experienced in daily life. Participants were asked to write down their hindering health complaints and additional information. For this, the questionnaire showed four boxes, which were numbered complaint 1 to complaint 4. Next to each of the four boxes was an additional box called ‘(optional) explanation’ (S1 Appendix). This open-ended question was not part of any of the previously mentioned domains.

### Coding strategy for open question about hindering complaints

All answers to the open-ended question were coded according to the Dutch version of the International Classification of Primary Care (ICPC-1-NL) [19–21]. This classification system is used by Dutch GPs. The ICPC-1-NL is categorised in 17 chapters according to the different organ systems (chapter B to N and R to Y). The exceptions are chapter A ‘General and unspecified’, which includes symptoms as pain and fever and diagnoses as fall/trauma/injury or Lyme’s disease, chapter P ‘Psychological problems’, and chapter Z ‘Social Problems’. Each chapter is further divided into different components, i.e. complaints/symptoms (01–29), diagnostic and preventive actions, treatment and therapeutic actions, test results, administrative actions and referrals or other reasons for the consultation (30–69), and diagnoses/diseases (70 and higher). An ICPC-code always consists of a letter (chapter) and numbers (component).

Since the aim of this study was to provide an overview of self-reported hindering complaints regardless of the possible presence of (chronic) diseases, we excluded from the analysis the items in which diagnoses/diseases were reported (n = 5091). For this purpose we classified some items in a different component than in the original ICPC-1-NL. Because the ICPC-1-NL did not provide suitable codes for some problems, we added codes for: pain at a specific site, problems with walking/standing, problems with manual skills, problems with riding a bike/driving/use of public transport, fear of falling, risk of falling, answers not referring to a problem, and answers we could not interpret. We used two separate codes for an accident and for a fall. The answers were coded by two authors (SCEB and OYAC) according to a pre-defined protocol. Disagreement was resolved by consensus. In case of no consensus a third author (JWB) was consulted.

### Functional outcomes

Socio-demographic characteristics and validated questionnaires on functional outcomes for a selection of the participants, as described earlier, were obtained during home visits by research nurses (Fig 1).

#### Number of domains with problems

When a participant had a positive response on two or more items in a domain on the ISCOPE screening questionnaire, this health domain was considered as a health domain with problems. When participants had problems on three or four domains according to the ISCOPE screening questionnaire, they were considered to have complex problems [18].

#### Activities of daily living (ADL)

The Groningen Activities Restriction Scale (GARS) was used to assess functioning in daily life. This questionnaire includes nine questions on Basic Activities of Daily Living (BADL) and nine questions on Instrumental Activities of Daily Living (IADL). For all questions participants could indicate whether they could do an activity independently. Answer categories are ‘yes, fully independent without any problems’ (1 point), ‘yes fully independent, but with some difficulty’ (2 points), ‘yes, fully independent, but with a lot of difficulty’ (3 points), ‘no, only with someone’s help’ (4 points).The total score ranges from 18 (no disability present) to 72 (highly disabled) [22].

#### Quality of life

QoL was measured with Cantril’s Ladder. Participants were asked to rate their experienced QoL on a scale ranging from 0 (low satisfaction) to 10 (high satisfaction) [23].

#### Cognitive function

The 30-item Minimal Mental State Examination (MMSE) was used to assess cognitive function (range 0–30). Lower scores indicate lower cognitive function [24].

#### Depressive symptoms

To assess depressive symptoms, the 15-item Geriatric Depression Scale (GDS-15) was used with participants with an MMSE score >18. For all items (positive and negative) participants were asked if they agreed with the statement. Participants received zero or one point per item. The total scores ranged from 0 (no complaints) to 15 (severe depressive symptoms) [25].

#### Loneliness

Loneliness was assessed with the Loneliness Scale of De Jong Gierveld (DJG), with six questions on emotional loneliness and five items on social loneliness (range 0–11), with a higher score indicating more severe experienced loneliness. For all items (positive and negative) participants were asked if they agreed with the statement. Participants received zero or one point per item. This questionnaire was used only with participants with an MMSE score >18 [26].

### Electronic Patient Register (EPR) of the general practices

The EPRs from one year before until one year after receiving the ISCOPE questionnaire were searched for participants who reported one or more of the three most reported hindering complaints in the ISCOPE screening questionnaire. Because not all complaints in the EPRs were coded with the appropriate ICPC-1-NL code, we searched the EPRs with keywords and synonyms to establish whether the top-3 of self-reported hindering complaints were mentioned. If a complaint was found in the EPR of a patient, we considered this complaint to be known by the GP. Since some electronic systems did not allow data extraction, these were not available for all participants.

### Statistical methods

Categorical data were described as proportions and compared by Chi-squared tests. Considering the skewed distribution of the data, the Mann-Whitney U-test and Jonckheere Terpstra test were used to compare the number of self-reported hindering complaints between two independent groups or for more than two independent, ordered alternatives, respectively. With linear regression analysis, adjusted for age and sex, we evaluated the association between the number of self-reported hindering complaints and the different outcome measurements. Besides the unstandardized coefficients, we also report the standardized coefficients in order to compare the effect of the number of self-reported hindering complaints between the different outcome measures.

The association between the ten most reported hindering complaints and the different outcome measurements were also evaluated with linear regression, adjusted for age and sex, with and without adjustment for the number of self-reported hindering complaints. To perform this analysis the independent variables were transformed to a dichotomous variable (complaint present or not).

All analyses were performed using SPSS 20 for Windows. A p-value of <0.05 was considered statistically significant.

## Results

Of the 11,476 older persons who received the postal ISCOPE screening questionnaire, 7285 gave informed consent and returned the completed questionnaire (response 63.5%). A total of 2713 participants (of which 17.1% had problems on 0–1 domain, 27.0% problems on 2 domains and 55.9% with problems on 3–4 domains) were visited at home (Fig 1). For practical reasons, it was not possible to extract the data from all the different EPRs. Full data from the EPR were available for 4360 participants.

The median age of the population was 81.0 (IQR 77.8–85.3) years; 38.6% of the participants (n = 2813) were male. 53.4% of the participants (n = 3890) reported the use of ≥4 different drugs a day. Of all participants, 2023 (27.8%) reported no problems on any domain of the ISCOPE screening questionnaire, and 1921 participants (26.4%) were considered to have complex problems (problems on ≥3 domains). Of all participants, 68.3% considered themselves able to cope with day-to-day life (Table 1).

**Table 1:** Baseline characteristics of the study population (n = 7285).

Characteristics		n	%
Age (years)			
	75–84	5369	73.7
	85–94	1804	24.8
	95–105	112	1.5
Sex			
	Male	2813	38.6
	Female	4472	61.4
Number of domains with problems (ISCOPE questionnaire)			
No complex problems		5357	73.5
	none	2023	27.8
	1 domain	1783	24.5
	2 domains	1551	21.3
Complex problem		1921	26.4
	3 domains	1281	17.6
	4 domains	640	8.8
Own opinion on coping with day-to-day life			
	Well	4977	68.3
	Average	1956	26.8
	Not at all well	341	4.7
Self-reported number of drugs used per day			
	Less than 4 drugs	3387	46.5
	4 or more drugs	3890	53.4

### Self-reported hindering complaints

The 7285 participants reported a total of 13,524 hindering complaints. The median number of self-reported hindering complaints per person was 1 (IQR 0–3, range 0–18). Of all participants, 2379 (32.7%) did not report any hindering complaints, 6.0% (n = 441) reported ≥6 complaints. Significantly higher numbers of self-reported hindering complaints were observed for women, with increasing age, with an increasing number of domains with problems, with worse coping with day-to-day life, and with ≥4 different drugs per day (p-values <0.001) (Table 2).

**Table 2:** Number of self-reported hindering complaints depending on patient characteristics in older persons, stratified for sex (n = 7285).

		Total (n = 7285)			Male (n = 2813)			Female (n = 4472)		
Patient characteristics		Median	IQR	P-value	Median	IQR	P-value	Median	IQR	P-value
Age (years)				< 0.001^b^			< 0.001^b^			< 0.001^b^
	75–84	1	0–3		1	0–3		1	0–3	
	85–94	2	0–3		2	0–3		2	1–3	
	95–105	2	1–4		3	1–4		2	1–4	
Sex				< 0.001^a^						
	Male	1	0–3							
	Female	1	0–3							
Number of domains with problems				< 0.001^b^			< 0.001^b^			< 0.001^b^
	None	0	0–1		0	0–1		0	0–1	
	1 domain	1	0–2		1	0–2		1	0–2	
	2 domains	2	1–3		2	1–3		2	1–3	
	3 domains	2	1–4		3	1–4		2	1–4	
	4 domains	3	1–5		3	1–4		3	1–5	
Own opinion on coping with day-to-day life				< 0.001^b^			< 0.001^b^			< 0.001^b^
	Good	1	0–2		1	0–2		1	0–2	
	Moderate	2	1–4		2	1–4		2	1–4	
	Bad	3	1–4		3	1–5		3	1–4	
Self-reported number of drugs used per day				< 0.001^a^			< 0.001^a^			< 0.001^a^
	Less than 4 drugs	1	0–2		1	0–2		1	0–2	
	4 or more drugs	2	1–3		2	0–3		2	1–3	

^a^ Mann-Whitney U

^b^Jonckheere-Terpstra

The top-10 of the most reported hindering complaints per person, stratified for sex, is listed in Table 3. Most participants reported problems with walking or standing (n = 1609, 22.1%) and pain (n = 1515, 20.8%), followed by weakness/tiredness (n = 620, 8.5%) and visual complaints (n = 549, 7.5%).

**Table 3:** Number of older persons with the top-10 of self-reported hindering complaints, stratified for sex (n = 7285).

Self-reported hindering complaints	Total (n = 7285)		Male (n = 2813)		Female (n = 4472)		P-value^a^
	n	%	n	%	n	%	
Limited walking/standing	1609	22.1	555	19.7	1054	23.6	< 0.001
Pain^b^	1515	20.8	429	15.3	1086	24.3	< 0.001
Weakness/tiredness	620	8.5	185	6.6	435	9.7	< 0.001
Visual complaints/limitations^c^	549	7.5	194	6.9	355	7.9	0.101
Hearing complaints/limitations^d^	423	5.8	192	6.8	231	5.2	0.003
Dyspnea because of respiratory tract	388	5.3	173	6.2	215	4.8	0.013
Back complaints/symptoms (not mentioning pain)	373	5.1	116	4.1	257	5.7	0.002
Cognitive complaints/symptoms	305	4.2	127	4.5	178	4.0	0.268
Other neurologic complaints/symptoms	278	3.8	119	4.2	159	3.6	0.143
Incontinence (urine/feces)^e^	268	3.7	89	3.2	179	4.0	0.064

^a^ Chi-square-test

^b^ Includes: pain; pain, specific sites; abdominal pain/cramps, general; abdominal pain, epigastric; rectal/anal pain; other localized abdominal pain; eye pain; ear pain; heart pain; other cardiovascular pain; low back pain without radiating pain; muscle pain; lower back pain with radiating pain; headache; pain face; migraine; cluster headache; pain because of airways; complaint/symptom sinus (including pain); sore throat; pain of the skin; post-herpetic neuralgia; painful urination; renal colic; genital pain (female); pain in breast (female) and pain in testis/scrotum.

^c^ Includes: visual complaints; disability/limitation because of the eye; limited riding a bike/driving/use of public transport because of problems eye and blindness, every type/degree.

^d^ Includes: hearing complaints; disability/limitation because of the ear; acoustic deafness and deafness.

^e^ Includes: urinary incontinence and incontinence of faeces.

### Associations between number and type of self-reported hindering complaints and functional outcomes

In the sample of participants with functional outcomes measured, a higher number of self-reported hindering complaints was associated with a higher number of domains with problems on the ISCOPE questionnaire and poorer scores on the GARS, GDS-15, DJG, and QoL measured on Cantril’s Ladder. The number of self-reported hindering complaints showed the strongest associations with the number of domains with problems, followed by poorer scores on the GARS, GDS-15, and QoL on Cantril’s Ladder. There was no statistically significant association between the number of self-reported hindering complaints and the MMSE score (Table 4).

**Table 4:** Association between number of self-reported hindering complaints and functional outcomes in older persons, crude and adjusted for age and sex.

		Crude				Adjusted^a^			
Outcome measurement	n	B	Beta	95% CI	P-value	B	Beta	95% CI	P-value
Domains with problems	7278	0.29	0.41	0.28 to 0.31	< 0.001	0.27	0.38	0.26 to 0.29	< 0.001
GARS	2687	1.25	0.19	1.01 to 1.49	< 0.001	1.07	0.17	0.85 to 1.29	< 0.001
QoL on Cantril's Ladder	2682	-0.13	-0.18	-0.15 to -0.10	< 0.001	-0.13	-0.18	-0.15 to -0.10	< 0.001
MMSE	2679	0.01	0.01	-0.06 to 0.08	0.741	0.04	0.02	-0.03 to 0.11	0.232
GDS-15	2552	0.26	0.18	0.20 to 0.31	< 0.001	0.25	0.18	0.20 to 0.30	< 0.001
DJG	2546	0.20	0.13	0.15 to 0.26	< 0.001	0.20	0.13	0.14 to 0.26	< 0.001

a Adjusted for age and sex

Abbreviations: GARS = Groningen Activities Restriction Scale; QoL = quality of life; MMSE = Mini Mental State Examination; GDS-15 = Geriatric Depression Scale– 15 items; DJG = De Jong Gierveld loneliness scale

Weakness, visual limitations, cognitive complaints and back complaints are associated with a higher number of domains with problems. Problems with walking/standing, visual limitations and dyspnea were all associated with poorer scores on ADL (GARS). Problems with walking/standing and cognitive problems were associated with lower cognitive functioning; weakness was associated with higher cognitive function (MMSE). Cognitive problems were associated with more depressive symptoms (GDS-15). Hearing complaints were associated with less experiences loneliness (DJG). We found no significant association between the ten most reported hindering complaints and QoL (Cantril’s Ladder). All results were adjusted for age, sex and the number of self-reported hindering complaints (S2 Appendix includes results with and without adjustment for the number of self-reported hindering complaints).

### Visits to the GP for the self-reported hindering complaints

Data from the EPRs were available for 874 of 1609 participants who reported problems with walking/standing; for 28.3% (n = 247), this complaint was mentioned in the EPR. Data from the EPR were available for 346 of the 620 participants who reported weakness/tiredness; for 55.5% (n = 192), this was mentioned in the EPR. Data from the EPR were available for 872 of the 1515 participants who reported pain; in 91.3% (n = 796) this was mentioned in the EPR.

## Discussion

The present study investigated hindering health complaints, self-reported by community-dwelling older persons. Large differences in number and type of complaints were observed. About one third of all participants did not report any complaints at all. The number of self-reported complaints ranged from 0 to18 and was significantly higher for women and in older age groups. Most older persons who reported complaints experienced problems with walking/standing, pain, or weakness/tiredness.

The number of self-reported hindering complaints was associated with lower scores on ADL, QoL, depression, loneliness, and with a higher number of domains with problems. The strongest association with the number of self-reported hindering complaints was found for the number of domains with problems. We found no association with cognitive function.

Specific self-reported hindering complaints such as weakness, problems with walking/standing, visual limitations, cognitive problems, dyspnea and back complaints were related to a higher number of domains with problems, poorer scores on ADL, cognitive function or depressive symptoms. There was no association with QoL.

When searching the data from the EPRs for the three most reported complaints, differences were found regarding the percentages of registration of the complaints in the EPR: e.g. pain was mentioned in 91.3% of the cases and problems with walking/standing in 28.3%.

### Strengths and limitations

An important strength of this study is that participants did not have to choose between options from a standard list of complaints, but (based on an open-ended question) could report all complaints that actually hindered them, which leaves room for unanticipated answers.

Moreover, we had the unique opportunity to investigate the self-reported hindering complaints in a large sample of older persons in primary care, and to link this information to scores on several validated questionnaires on functional outcomes and to information in the EPRs. To our knowledge this is the first study of this size to investigate the association between self-reported hindering complaints, functional outcomes, and the EPR of the GP in this older population.

Although we purposefully chose the methodology, use of an open-ended question to determine the number of experienced complaints is open to debate. Previous studies on self-reported conditions showed that about 50% of all reported conditions was mentioned after asking an initial open-ended question. Over a third of the total conditions was reported only after showing participants a card with several examples of conditions listed [27]. This suggests that the number of hindering complaints we found is an underestimation of the true number of complaints. However, this problem is probably less pronounced in the present study because we were specifically interested in self-reported hindering complaints. We believe that patients might be more aware of, and more likely to report complaints that hinder them every day than conditions that might not bother them, or only to a small extent.

A limitation of this study is that the responses to the open-ended question might be influenced by closed-ended questions posed earlier in the questionnaire. These questions could have reminded participants of complaints, or have given them the impression that it was unnecessary to report complaints a second time. Furthermore, an open-ended question carries the risk of possible misinterpretation of the responses and possible problems with coding the self-reported hindering complaints.

Finally, not all complaints discussed with the GP are necessarily registered in the EPR. Therefore, the percentages of complaints reported in the EPR have to be interpreted with caution.

### Comparison with previous research

Our study builds on results from previous studies reporting on health complaints in the older population. The Dutch NIVEL database consists of data from EPRs from general practices (including ≥385,000 patients of all ages) and data from a questionnaire among the Dutch population (~2,000 persons aged ≥65 years). In the NIVEL study, the most prevalent reported complaints for persons aged ≥65 years were lower back pain, fatigue/weakness, sleeping problems, neck or shoulder pain, and hearing problems. Our study confirms that pain, weakness/tiredness and hearing problems are among the most reported complaints by older persons. In contrast with our study, the NIVEL study does not report problems with walking/standing as one of the most reported problems. However, it must be noted that this difference is probably due to the differences in methods between the studies, since problems with walking/standing were not on the list of most prevalent complaints in the NIVEL questionnaires [28, 29].

A Swedish study among 448 community-dwelling older persons (who received help with ADL), found pain and impaired mobility as the most frequently reported complaints on a standard list over a 3-month period. The number of complaints (range 0–26, median 10) was associated with a lower QoL [30].

An Italian study among 747 community-dwelling older persons reported depression, pain, and anxiety as the most prevalent reported complaints (median 6) in a standard list. The authors also showed that the combined presence of fatigue, memory loss, nutrition, indigestion, hearing, and speaking was associated with psychological distress, mental impairment and ADL dependency [31].

Several limitations arise when comparing the results of these earlier studies with the present study, mainly due to possible differences in socio-economic conditions, and in study design, and because of the inclusion of specific complaints and diagnoses in these earlier studies, whereas we focused only on self-reported complaints which caused hindrance in daily life. Also, earlier studies presented lists of complaints from which participants had to choose [28–31]. Although this avoids misinterpretation of the answers, it leaves little room for unanticipated answers. Finally, the methods used to measure QoL and functional status differed. Nevertheless, despite all differences, we conclude that our study confirms the high prevalence of pain, weakness/tiredness and mobility problems, as well as the association between complaints and QoL and functioning.

### Implications for clinical practice

First, it is important to note that, in our study population of persons aged ≥75 years, about one third reported no hindering complaints. For the remaining two thirds who did experience hindering complaints, it could be important for GPs to discuss these complaints and options for intervention with their patients in order to prevent (further) disability and dependency. Regularly asking older patients about these hindering complaints can provide the GP with extra information about problems experienced daily and can be used to give appropriate advice. Problems concerning walking/standing and pain are most often reported. We show that some of the ten most reported hindering complaints are associated with poorer scores on functional outcomes. These findings add to the growing awareness that it is important to recognize these complaints in an early stage. However, there are some unexpected findings as the association between weakness and a better cognitive function and hearing complaints and less loneliness. These can be coincidental findings because of multiple testing. A proactive attitude of the GP may reveal these hindering complaints at an earlier stage and could possibly open new ways for intervention. Although it is not always possible to solve the underlying pathology of functional limitations in older persons, use of a functional approach to their complaints might allow improvements in functioning to be made [32].

Previous research has shown that more attention should be paid to the functional status of older persons and to problems hindering them in maintaining their independency [8–14]. To achieve this, healthcare providers and patients have to make a transition from disease-oriented, reactive healthcare towards functional-oriented, proactive healthcare [3, 4]. The knowledge on self-reported hindering complaints acquired during the present study can be used by GPs to further shape this transition and to structure and optimize the necessary advice and treatment.

### Future research

As expected, for those cases in which one of the top-3 complaints had been reported, this complaint was not always mentioned in the EPR. This might be because not all GPs consistently register all complaints mentioned during a consultation. However, the differences found are striking: e.g. problems with walking/standing were found in the EPR in only 28.3% of the cases whereas pain was mentioned in 91.3% of the cases. Further research is needed to elucidate the reasons behind this large difference.

Although a spectrum of self-reported hindering complaints of older persons and the association with functional outcomes has emerged from this study, it remains unclear whether advice and/or treatment from the GP or from another medical professional is needed. Future studies should focus on the wishes and needs of older persons related to these problems and on the effect of pro-active treatment of these problems on the disability and dependency of older persons.

## Supporting Information

S1 Appendix ISCOPE screening questionnaire. (DOCX)

S2 Appendix Association between the 10 most reported hindering complaints and functional outcomes in older persons adjusted for age and sex and with and without adjustment for the number of self-reported hindering complaints. (DOCX)
