# Supplementary material for: Self-Reported Hindering Health Complaints of Community-Dwelling Older Persons: A Cross-Sectional Study
Source: PLoS One. 2015 Nov 16;10(11):e0142416. doi: 10.1371/journal.pone.0142416 (PMC4646486; doi:10.1371/journal.pone.0142416)
Supplement: S1 Appendix — (DOCX) [file pone.0142416.s001.docx]

**Appendix 1. ISCOPE screening questionnaire**

**Daily life abilities**

These first questions relate to how you function/manage day-to-day life.

You may be helped in these activities by aids such as a stick walking frame or wheelchair.

1. Can you do the shopping without help from anyone else?

*Yes / No*

2. Can you walk outdoors without help from anyone else?

*Yes / No*

3. Can you dress and undress yourself without help from anyone else?

*Yes / No*

4. Can you go to the toilet without help from anyone else?

*Yes / No*

5. Can you manage your finances yourself (collect your money, pay your bills)?

*Yes / No*

6. How well would you say you cope with your general day-to-day life?

*Well / Average /Not at all well*

**Health and illness**

7. Which mark would you give for your physical fitness?

*1 2 3 4 5 6 7 8 9 10*

*Not at all fit Very fit*

8. Do you experience day-to-day problems due to poor eyesight (even if you wear glasses or contact lenses)?

*Yes / No*

9. Do you experience day-to-day problems due to poor hearing (even if you wear a hearing aid)?

*Yes / No*

10. Do you experience problems with incontinence of urine or stool?
*Yes / No*

11. Do you experience daily problems due to pain?
*Yes / No*

12. Have you lost weight (more than 6 kg) in the last 6 months unintentionally?

*Yes / No*

13. Are you using more than 4 different kinds of medicine at the moment?

*Yes / No*

14. Have you had a fall in the last month?

*Yes / No*

15. Have you been admitted to the hospital in the last 6 months?

*Yes / No*

**Psychological functioning**

16. Do you feel you have memory complaints?

*Yes /Sometimes/ No*

17. Have you recently felt sad or depressed?

*Yes /Sometimes/ No*

18. Have you recently felt nervous or anxious?

*Yes /Sometimes/ No*

19. Do you feel pretty worthless at the moment?

*Yes /Sometimes/ No*

**Social functioning**

20. Do you feel that your life is empty?

*Yes /Sometimes/ No*

21. Do you feel the lack of a close friend?

*Yes /Sometimes/ No*

22. Do you feel left alone sometimes?

*Yes /Sometimes/ No*

23. Do you feel there are enough people with whom you feel a close connection?

*Yes /Sometimes/ No*

24. Do you receive help from anybody in you immediate surrounding because you are unable to do things for yourself?
*Yes / No*

25. Has anyone helped you to fill in this questionnaire?
*No, I have filled in the questionnaire myself.
Yes, someone helped me to answer these questions.
Somebody has answered them for me.*

26. At the moment, which health complaints limit you the most in your day-to-day life?

Complaint 1 (Possible) Explanation

Complaint 2 (Possible) Explanation

Complaint 3 (Possible) Explanation

Complaint 4 (Possible) Explanation
