# Supplementary material for: Self-Reported Hindering Health Complaints of Community-Dwelling Older Persons: A Cross-Sectional Study
Source: PLoS One. 2015 Nov 16;10(11):e0142416. doi: 10.1371/journal.pone.0142416 (PMC4646486; doi:10.1371/journal.pone.0142416)
Supplement: S2 Appendix — (DOCX) [file pone.0142416.s002.docx]

| **Table 5. Association between the 10 most reported hindering complaints and functional outcomes in older persons** | | | | | | | | | |
| --- | --- | --- | --- | --- | --- | --- | --- | --- | --- |
| **adjusted for age and sex^a^ and adjusted for age, sex and the number of self-reported hindering problems^b^** | | | | | | | | |  |
|  |  |  | Outcome measurement | | | |  | |  |
|  | Domains with problems^a^ (n=7278) | | |  | Domains with problems^b^ (n=7278) | | | | |
| Self-reported hindering complaints | B | 95% CI | P-value |  | B | 95% CI | | P-value | |
| Pain | 0.53 | 0.46 to 0.60 | <0.001 |  | 0.01 | -0.06 to 0.09 | | 0.697 | |
| Limited walking/standing | 0.57 | 0.50 to 0.64 | <0.001 |  | 0.07 | -0.00 to 0.14 | | 0.054 | |
| Weakness/tiredness | 0.72 | 0.62 to 0.82 | <0.001 |  | 0.20 | 0.10 to 0.29 | | <0.001 | |
| Incontinence (urine/feces) | 0.39 | 0.24 to 0.54 | <0.001 |  | -0.12 | -0.26 to 0.03 | | 0.113 | |
| Hearing complaints/limitations | 0.50 | 0.38 to 0.62 | <0.001 |  | 0.02 | -0.10 to 0.13 | | 0.805 | |
| Visual complaints/limitations | 0.71 | 0.60 to 0.82 | <0.001 |  | 0.29 | 0.18 to 0.39 | | <0.001 | |
| Cognitive complaints/symptoms | 0.74 | 0.60 to 0.88 | <0.001 |  | 0.30 | 0.16 to 0.43 | | <0.001 | |
| Dyspnea because of respiratory tract | 0.56 | 0.43 to 0.68 | <0.001 |  | 0.10 | -0.02 to 0.22 | | 0.096 | |
| Back complaints/symptoms (not mentioning pain) | 0.17 | 0.05 to 0.30 | 0.008 |  | -0.19 | -0.31 to -0.07 | | 0.002 | |
| Other neurologic complaints/symptoms | 0.61 | 0.46 to 0.75 | <0.001 |  | 0.08 | -0.06 to 0.22 | | 0.241 | |
|  |  |  |  |  |  |  | |  | |
|  | GARS^a^ (n=2687) | | |  | GARS^b^ (n=2687) | | | | |
| Self-reported hindering complaints | B | 95% CI | P-value |  | B | 95% CI | | | P-value |
| Pain | 2.23 | 1.27 to 3.20 | <0.001 |  | 0.37 | -0.68 to 1.42 | | | 0.493 |
| Limited walking/standing | 5.78 | 4.86 to 6.71 | <0.001 |  | 4.73 | 3.71 to 5.75 | | | <0.001 |
| Weakness/tiredness | 0.92 | -0.37 to 2.20 | 0.163 |  | -0.93 | -2.25 to 0.40 | | | 0.170 |
| Incontinence (urine/feces) | 2.94 | 0.90 to 4.98 | 0.005 |  | 1.21 | -0.83 to 3.26 | | | 0.244 |
| Hearing complaints/limitations | 1.17 | -0.51 to 2.85 | 0.173 |  | -0.79 | -2.50 to 0.92 | | | 0.363 |
| Visual complaints/limitations | 2.83 | 1.42 to 4.24 | <0.001 |  | 1.45 | 0.02 to 2.87 | | | 0.046 |
| Cognitive complaints/symptoms | 3.13 | 1.23 to 5.03 | 0.001 |  | 1.70 | -0.20 to 3.60 | | | 0.079 |
| Dyspnea because of respiratory tract | 3.23 | 1.55 to 4.90 | <0.001 |  | 1.87 | 0.20 to 3.55 | | | 0.029 |
| Back complaints/symptoms (not mentioning pain) | -0.35 | -2.15 to 1.45 | 0.704 |  | -1.45 | -3.24 to 0.34 | | | 0.112 |
| Other neurologic complaints/symptoms | 1.47 | -0.40 to 3.35 | 0.122 |  | -0.18 | -2.05 to 1.70 | | | 0.854 |
|  |  |  |  |  |  |  | | |  |
|  | Qol on Cantril's Ladder^a^ (n=2682) | | |  | Qol on Cantril's Ladder^b^ (n=2682) | | | | |
| Self-reported hindering complaints | B | 95% CI | P-value |  | B | 95% CI | | | P-value |
| Pain | -0.33 | -0.44 to -0.21 | <0.001 |  | -0.12 | -0.25 to 0.00 | | | 0.056 |
| Limited walking/standing | -0.25 | -0.36 to -0.14 | <0.001 |  | -0.04 | -0.16 to 0.09 | | | 0.578 |
| Weakness/tiredness | -0.25 | -0.40 to -0.10 | 0.001 |  | -0.05 | -0.21 to 0.11 | | | 0.546 |
| Incontinence (urine/feces) | 0.02 | -0.22 to 0.26 | 0.865 |  | 0.24 | -0.01 to 0.48 | | | 0.058 |
| Hearing complaints/limitations | -0.15 | -0.35 to 0.05 | 0.153 |  | 0.08 | -0.12 to 0.28 | | | 0.436 |
| Visual complaints/limitations | -0.28 | -0.45 to -0.12 | 0.001 |  | -0.12 | -0.29 to 0.05 | | | 0.163 |
| Cognitive complaints/symptoms | -0.10 | -0.32 to 0.13 | 0.410 |  | 0.08 | -0.15 to 0.31 | | | 0.480 |
| Dyspnea because of respiratory tract | -0.19 | -0.39 to 0.01 | 0.059 |  | -0.03 | -0.22 to 0.17 | | | 0.806 |
| Back complaints/symptoms (not mentioning pain) | 0.02 | -0.20 to 0.23 | 0.889 |  | 0.15 | -0.07 to 0.36 | | | 0.177 |
| Other neurologic complaints/symptoms | -0.33 | -0.55 to -0.11 | 0.003 |  | -0.15 | -0.37 to 0.08 | | | 0.199 |
|  |  |  |  |  |  |  | | |  |
|  |  |  |  |  |  |  | | |  |
|  | MMSE^a^ (n=2679) | | |  | MMSE^b^ (n=2679) | | | | |
| Self-reported hindering complaints | B | 95% CI | P-value |  | B | 95% CI | | | P-value |
| Pain | 0.03 | -0.27 to 0.33 | 0.846 |  | -0.06 | -0.39 to 0.27 | | | 0.730 |
| Limited walking/standing | -0.30 | -0.59 to -0.00 | 0.047 |  | -0.45 | -0.77 to -0.13 | | | 0.006 |
| Weakness/tiredness | 0.71 | 0.32 to 1.11 | <0.001 |  | 0.70 | 0.29 to 1.11 | | | 0.001 |
| Incontinence (urine/feces) | 0.24 | -0.38 to 0.87 | 0.444 |  | 0.18 | -0.46 to 0.82 | | | 0.578 |
| Hearing complaints/limitations | -0.23 | -0.75 to 0.28 | 0.377 |  | -0.33 | -0.86 to 0.21 | | | 0.228 |
| Visual complaints/limitations | -0.35 | -0.78 to 0.09 | 0.119 |  | -0.42 | -0.86 to 0.02 | | | 0.063 |
| Cognitive complaints/symptoms | -3.73 | -4.30 to -3.17 | <0.001 |  | -3.90 | -4.47 to -3.33 | | | <0.001 |
| Dyspnea because of respiratory tract | -0.24 | -0.75 to 0.28 | 0.361 |  | -0.31 | -0.83 to 0.22 | | | 0.251 |
| Back complaints/symptoms (not mentioning pain) | 0.43 | -0.12 to 0.98 | 0.122 |  | 0.40 | -0.16 to 0.95 | | | 0.161 |
| Other neurologic complaints/symptoms | 0.59 | 0.02 to 1.16 | 0.044 |  | 0.54 | -0.41 to 1.13 | | | 0.069 |
|  |  |  |  |  |  |  | | |  |
|  | GDS-15^a^ (n=2552) | | |  | GDS-15^b^ (n=2552) | | | | |
| Self-reported hindering complaints | B | 95% CI | P-value |  | B | 95% CI | | | P-value |
| Pain | 0.55 | 0.32 to 0.78 | <0.001 |  | 0.12 | -0.13 to 0.37 | | | 0.350 |
| Limited walking/standing | 0.29 | 0.06 to 0.52 | 0.014 |  | -0.18 | -0.43 to 0.07 | | | 0.158 |
| Weakness/tiredness | 0.46 | 0.16 to 0.77 | 0.003 |  | 0.06 | -0.26 to 0.37 | | | 0.725 |
| Incontinence (urine/feces) | 0.33 | -0.17 to 0.82 | 0.194 |  | -0.11 | -0.60 to 0.39 | | | 0.669 |
| Hearing complaints/limitations | 0.36 | -0.06 to 0.77 | 0.090 |  | -0.09 | -0.50 to 0.33 | | | 0.682 |
| Visual complaints/limitations | 0.60 | 0.25 to 0.94 | 0.001 |  | 0.28 | -0.06 to 0.63 | | | 0.106 |
| Cognitive complaints/symptoms | 0.87 | 0.37 to 1.36 | 0.001 |  | 0.52 | 0.03 to 1.02 | | | 0.037 |
| Dyspnea because of respiratory tract | 0.26 | -0.15 to 0.66 | 0.215 |  | -0.08 | -0.48 to 0.33 | | | 0.703 |
| Back complaints/symptoms (not mentioning pain) | 0.03 | -0.40 to 0.45 | 0.900 |  | -0.24 | -0.66 to 0.19 | | | 0.273 |
| Other neurologic complaints/symptoms | 0.59 | 0.14 to 1.03 | 0.010 |  | 0.22 | -0.23 to 0.67 | | | 0.330 |
|  |  |  |  |  |  |  | | |  |
|  |  |  |  |  |  |  | | |  |
|  | DJG^a^ (n=2546) | | |  | DJG^b^ (n=2546) | | | | |
| Self-reported hindering complaints | B | 95% CI | P-value |  | B | 95% CI | | | P-value |
| Pain | 0.46 | 0.21 to 0.71 | <0.001 |  | 0.12 | -0.16 to 0.39 | | | 0.411 |
| Limited walking/standing | 0.26 | 0.01 to 0.51 | 0.042 |  | -0.11 | -0.38 to 0.16 | | | 0.428 |
| Weakness/tiredness | 0.16 | -0.17 to 0.49 | 0.354 |  | -0.19 | -0.53 to 0.15 | | | 0.274 |
| Incontinence (urine/feces) | 0.39 | -0.15 to 0.92 | 0.155 |  | 0.04 | -0.50 to 0.59 | | | 0.873 |
| Hearing complaints/limitations | -0.12 | -0.57 to 0.32 | 0.589 |  | -0.51 | -0.96 to -0.05 | | | 0.028 |
| Visual complaints/limitations | 0.11 | -0.26 to 0.48 | 0.559 |  | -0.15 | -0.53 to 0.22 | | | 0.423 |
| Cognitive complaints/symptoms | 0.34 | -0.20 to 0.88 | 0.213 |  | 0.05 | -0.49 to 0.59 | | | 0.850 |
| Dyspnea because of respiratory tract | 0.10 | -0.33 to 0.54 | 0.641 |  | -0.17 | -0.61 to 0.27 | | | 0.452 |
| Back complaints/symptoms (not mentioning pain) | -0.10 | -0.56 to 0.36 | 0.669 |  | -0.31 | -0.78 to 0.15 | | | 0.183 |
| Other neurologic complaints/symptoms | 0.39 | -0.09 to 0.88 | 0.112 |  | 0.09 | -0.39 to 0.58 | | | 0.706 |
